# Brain insulin resistance and neuropsychiatric symptoms in Alzheimer’s disease: A role for dopamine signaling

**DOI:** 10.4103/NRR.NRR-D-25-00281

**Published:** 2025-04-29

**Authors:** Anastasia Kontogianni, Hongbin Yang, Wenqiang Chen

**Affiliations:** King’s College London, Institute of Psychiatry, Psychology and Neuroscience, London, UK; Department of Affiliated Mental Health Center of Hangzhou Seventh People’s Hospital, Liangzhu Laboratory, The State Key Lab of Brain-Machine Intelligence, Zhejiang University, Hangzhou, Zhejiang Province, China; MOE Frontier Science Center for Brain Science & Brain-Machine Integration, School of Brain Science and Brain Medicine, Zhejiang University, Hangzhou, Zhejiang Province, China; NHC and CAMS Key Laboratory of Medical Neurobiology, Zhejiang University, Hangzhou, Zhejiang Province, China; Steno Diabetes Center Copenhagen, Herlev, Denmark; Joslin Diabetes Center, Harvard Medical School, Boston, MA, USA

**Type 2 diabetes mellitus has central complications:** Diabetes, a metabolic disorder primarily characterized by hyperglycemia due to insufficient insulin secretion, or impaired insulin signaling, has significant central complications. Type 2 diabetes mellitus (T2DM), the most prevalent type of diabetes, affects more than 38 million individuals in the United States (approximately 1 in 10) and is defined by chronic hyperglycemia and insulin resistance, which refers to a reduced cellular response to insulin. While T2DM is commonly associated with peripheral complications, it also contributes to central complications, including neuropsychiatric symptoms (NPS) and cognitive decline (Chen et al., 2022), significantly accelerating the progression to Alzheimer’s disease (AD) and related dementias. At the core of these effects lies brain insulin resistance (BIR), a disruption in insulin signaling within the central nervous system that can occur even in individuals without diabetes (Chen et al., 2022).

**AD and the dopamine system:** AD, the leading cause of death among adults aged 65 and older, is a devastating neurodegenerative condition characterized by accumulation of amyloid-β (Aβ) plaques, tau hyperphosphorylation, and neuroinflammation. These pathological changes result in neuronal loss, synaptic dysfunction, and severe memory deficits.

**Figure 1:**
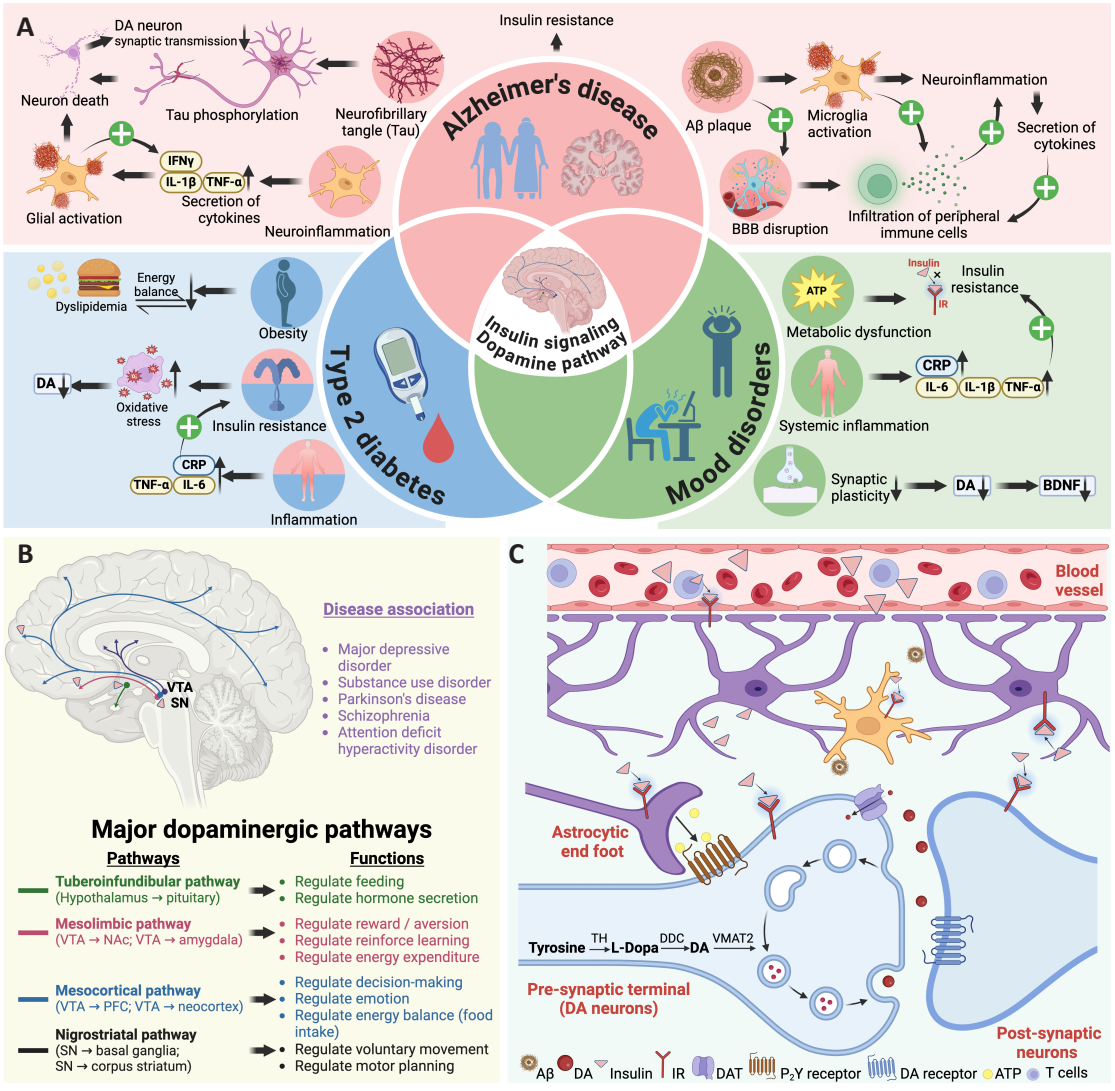
Dopaminergic system and its role in Alzheimer’s disease, type 2 diabetes, and mood disorders. (A) Interplay between DA pathway, AD, T2DM, and mood disorders. DA dysregulation is linked to AD (Aβ, tau, and neuroinflammation), T2D (insulin resistance and chronic inflammation), and mood disorders (metabolic dysfunction and synaptic plasticity loss). Shared mechanisms suggest DA and insulin as a key therapeutic target. (B) Key dopaminergic pathways. DA regulates feeding, hormone secretion, reward, decision-making, and movement via four key pathways: tuberoinfundibular, mesolimbic, mesocortical, and nigrostriatal pathways. Dysfunctions of these pathways are linked to many brain disorders including depression, Parkinson’s disease, schizophrenia, and ADHD. Pink triangles indicate prominent sites of insulin action in the brain. (C) Insulin regulates the synaptic transmission of dopaminergic neurons. DA is synthesized via TH and DDC, stored in VMAT2, and released to act on DA receptors. DA is cleared by DAT and degraded by MAO/COMT. Insulin signaling and neuroinflammatory factors (Aβ and infiltration of T cells) can modulate dopaminergic function. Created with BioRender.com. Aβ: Amyloid-beta; BBB: blood–brain barrier; BDNF: brain-derived neurotrophic factor; COMT: catechol-O-methyltransferase; CRP: C-reactive protein; DA: dopamine; DAT: dopamine transporter; DDC: dopamine decarboxylase; IFN-γ: interferon gamma; IL-1β: interleukin-1 beta; IR: insulin receptor; MAO: monoamine oxidase; NAc: nucleus accumbens; PFC: prefrontal cortex; SN: substantia nigra; T2D: type 2 diabetes; TH: tyrosine hydroxylase; TNF-α: tumor necrosis factor-alpha; VMAT2: vesicular monoamine transporter 2; VTA: ventral tegmental area.

Growing evidence suggests that insulin resistance plays a central role in bridging metabolic dysfunction with neurodegeneration and NPS in AD (**Figure 1A**). Although primarily recognized as a peripheral feature of T2DM, insulin resistance also affects the brain, known as BIR, which contributes to AD pathology (Chen et al., 2022). Notably, BIR disrupts dopamine (DA) neurotransmission (Kleinridders et al., 2015), a key regulator of motivation, reward, and mood (Zhou et al., 2024), linking metabolic dysfunction to both cognitive decline and NPS in AD. Understanding these mechanisms could open new therapeutic avenues for targeting insulin pathways to mitigate both cognitive impairment and NPS in AD.

While NPS encompass a broad spectrum of interchangeable symptoms, including agitation and aggression, this perspective focuses on mood disturbance, not only due to their strong link to insulin resistance and DA dysfunction, but also their significant impact on life quality and AD progression.

**Mood disturbance in T2DM and AD:** While mood disturbances are highly prevalent in both T2DM and AD, the biological mechanisms underlying these symptoms remain underexplored. Emerging evidence suggests that insulin resistance contributes to mood disorders by disrupting immuno-metabolic homeostasis, promoting neuroinflammation, and altering DA signaling, all of which are key factors implicated in the etiology of major depressive disorder (MDD). Many of these symptoms, including anhedonia and low motivation, stem from disruptions in DA signaling, which is highly sensitive to insulin action. Insulin resistance in brain regions, such as the ventral striatum and prefrontal cortex, impairs DA neurotransmission, reducing reward sensitivity and motivation, hallmarks of NPS observed in AD and T2DM.

Although AD is primarily associated with cognitive decline, NPS are also common as the disease advances. Many individuals with dementia or T2DM experience these disturbances (Chen et al., 2022), with their severity influenced by disease duration and cognitive decline (**Figure 1A**). Such symptoms contribute to increased mortality and place a significant burden on healthcare systems, particularly as more severe manifestations like psychosis and agitation often require pharmacological intervention (Chen et al., 2022). As AD progresses, NPS often intensify, in rare cases escalating to violence and self-harm. Thus, these challenges not only pose serious risks to the affected individuals but also impose substantial burdens on caregivers and the broader community. While drug treatments are available, their efficacy is limited, and concerns remain regarding the safety of antipsychotic medications. This highlights the urgent need to better understand the mechanisms driving NPS onset in T2DM and AD patients, to develop more effective and safer treatment strategies.

**Etiology of major depressive disorder: focus on immuno-metabolic pathways:** Mood disorders, including MDD being one of the most prevalent forms, are primarily characterized by disturbances in mood and motivation. Given the critical role of DA in reward processing and motivation, its dysfunction is a key contributor to depression. BIR further exacerbates mood disturbances by impairing DA signaling and promoting neuroinflammation, linking metabolic dysfunction to psychiatric symptoms. Hippocampal-specific insulin resistance has been shown to elicit mood dysfunction, including behavioral despair and anxiety-like phenotypes, independent of peripheral metabolic changes (Reagan et al., 2021).

Growing evidence suggests that MDD, as a multifactorial and highly heterogeneous disorder, is associated with immuno-metabolic dysfunction. This hypothesis proposes that systemic low-grade inflammation, primarily marked by elevated C-reactive protein, interleukin-6, and tumor necrosis factor-alpha, along with metabolic disturbances, such as insulin and leptin resistance, dyslipidemia and obesity, disrupts energy balance and DA-mediated reward processing (**Figure 1A**). These disruptions contribute to the characteristic symptoms of MDD, including anhedonia, hypersomnia, hyperphagia, and low motivation (Penninx et al., 2025).

Neuroinflammation plays a central role in this process, as pro-inflammatory cytokines from the periphery infiltrate the blood-brain barrier, activating microglia and astrocytes and triggering a neuroimmune response. Post-mortem studies of individuals with depression, particularly those who died by suicide, have revealed increased neuroinflammation, including elevated microglial density and higher levels of pro-inflammatory cytokines and quinolinic acid in brain regions critical for mood regulation (Steiner et al., 2011). Consistent with these findings, neuroimaging studies show that experimentally induced inflammation alters activity in the insula and ventral striatum, linking immune activation to dysconnectivity in mood-related brain circuits (Penninx et al., 2025). In parallel, preclinical models have demonstrated that systemic inflammation induced by lipopolysaccharide leads to persistent neuroinflammation and kynurenine pathway dysregulation, resulting in sustained depressive-like behaviors even after acute sickness resolves (Henry et al., 2008). Importantly, this chronic neuroinflammatory state also induces BIR, further impairing metabolic processes and disrupting mood-related circuits (Penninx et al., 2025).

BIR disrupts brain metabolism, altering homeostasis and energy balance. Leptin and insulin, key regulators of energy balance, fail to properly activate hypothalamic circuits, impairing their key functions, such as appetite regulation and energy sensing. This dysfunction extends to the ventral striatum, where midbrain DA projection terminals are located. Here, BIR reduces DA availability and blunts reward sensitivity, exacerbating symptoms of anhedonia, fatigue, and low motivation (Chen et al., 2022). Given that DA dysfunction is implicated in both depression and metabolic disorders, further examining the interaction between insulin signaling and DA pathways is critical for understanding how metabolic disturbances contribute to NPS.

**Interaction of DA system and insulin signaling in the brain:** Given the essential role of DA in motivation, reward processing, and mood regulation (Zhou et al., 2024), BIR profoundly alters DA neurotransmission, further linking metabolic dysfunction to mood disturbances (**Figure 1B** and **C**). Thus, investigating how insulin signaling interacts with the DA system is crucial for understanding the NPS observed in AD.

Insulin acts on dopaminergic neurons to increase DA release in the nucleus accumbens (NAc), which is mediated through the activation of striatal cholinergic interneurons (Stouffer et al., 2015). Using conditional knockout mice, our laboratory has previously shown that deficiency of insulin signaling in astrocytes leads to a 50% reduction in evoked DA release in the NAc and dorsal striatum, leading to the occurrence of behavioral deficits resembling mood disturbance in mice (Cai et al., 2018).

However, DA neurons comprise multiple neural subpopulations and form different DA pathways. For instance, the traditionally recognized mesolimbic DA pathway alone includes at least two distinct information processing pathways. We previously demonstrated that NAc subregions differentially regulate mesolimbic DA neurons through distinct inhibitory circuits, with medial shell neurons (NAc^Med^) directly inhibiting DA populations via direct GABA afferents, while lateral shell neurons (NAc^Lat^) primarily disinhibit DA neurons through VTA GABA interneurons (Yang et al., 2018). These findings highlight the role of NAc subregion-specific inputs in modulating DA activity and motivated behaviors. Importantly, insulin receptors are expressed in DA neurons and insulin signaling modulates DA release and plasticity (Kleinridders et al., 2015; Stouffer et al., 2015). Future studies should determine how BIR differentially affects DA subpopulations or DA pathways, further clarifying its role in NPS and AD.

**Brain insulin signaling in mood regulation and neurodegeneration:** Dysregulated insulin signaling not only disrupts DA homeostasis but also contributes to broader neurodegenerative processes, including synaptic dysfunction, oxidative stress, and neuroinflammation (**Figure 1A**). Given these widespread effects, BIR is increasingly recognized as a central factor in both mood regulation and neurodegenerative diseases such as AD.

In the healthy brain, insulin binds to insulin receptors expressed by various brain cell types, triggering mechanisms that regulate cellular processes such as oxidative stress, inflammation, synaptic plasticity, and cell survival (Chen et al., 2022). While insulin resistance is well characterized in peripheral tissues, its role in the brain has only recently gained attention, particularly in relation to AD and NPS.

We (the laboratory of Dr. Ronald Kahn) previously generated brain-specific IR knockout mouse models (Bruning et al., 2000), revealing key actions of insulin signaling in mood and cognition regulation. Subsequent studies have shown that BIR alters DA turnover, leading to behavioral impairment including anxiety- and depressive-like alterations (Kleinridders et al., 2015). In astrocytes, we have demonstrated that insulin regulates ATP exocytosis, whereas disruption of astrocytic insulin signaling reduces DA release in the forebrain, impacting mood and behavior (Cai et al., 2018). When insulin signaling is disrupted in microglia, mice exhibit social deficits and mood disturbances (Chen et al., 2024). At a cellular level, this is accompanied by alterations in cellular metabolism and activation of innate immune pathways. Collectively, these findings demonstrate the critical role of BIR in the development of mood disorders.

NPS in AD often worsens as the disease progresses, with evidence showing that AD pathology can exacerbate these symptoms. More recently, we began investigating impaired brain insulin signaling in neurodegeneration. Our findings indicate that the loss of astrocytic insulin signaling exacerbates AD-like phenotypes in an early-onset AD mouse model (5×FAD) (Chen et al., 2023). Importantly, these mice showed exacerbation of behavioral impairments. When insulin signaling is disrupted in microglia, microglial uptake of Aβ is impaired, accompanied by a worsening AD-related phenotype (Chen et al., 2024). These findings highlight the importance of brain insulin signaling in maintaining brain homeostasis and suggest its potential as a therapeutic target for reducing NPS in AD.

**Insulin action on synaptic transmission:** Given the essential role of insulin in maintaining neuronal function, disruptions in insulin signaling can impair synaptic plasticity and neurotransmission (**Figure 1C**). These synaptic deficits, particularly in glutamatergic and dopaminergic pathways, exacerbate cognitive and neuropsychiatric impairments in AD and T2DM.

Impaired brain insulin signaling disrupts synaptic transmission, a process critical for neuronal communication, plasticity, and cognitive function. Insulin plays a crucial role in modulating both excitatory and inhibitory synaptic activity, particularly in brain regions implicated in the NPS of AD. In the hippocampus, insulin resistance has been shown to cause dendritic atrophy, contributing to deficits in mood disturbance in rats (Reagan et al., 2021). In an animal model of high-fat diet-induced insulin resistance, impaired insulin signaling activates FoxO3a, disrupting AMPA receptor trafficking and weakening synaptic plasticity, leading to cognitive impairments (Spinelli et al., 2017).

Beyond the hippocampus, insulin also modulates dopaminergic signaling. In the VTA, insulin enhances NMDA receptor-mediated currents, increasing DA neuron excitability. In the NAc, insulin facilitates action potential-dependent DA release by activating striatal cholinergic interneurons, which stimulate nicotinic acetylcholine receptors on DA terminals, playing a key role in motivation and reward processing. However, in insulin resistance states (e.g., obesity and T2DM), this effect is impaired, potentially contributing to anhedonia and neuropsychiatric symptoms (Stouffer et al., 2015).

**Concluding remarks:** Insulin resistance plays a pivotal role in both cognitive decline and NPS by disrupting DA signaling and synaptic transmission. Restoring brain insulin sensitivity through intranasal insulin, insulin-sensitizing agents, anti-neuroinflammation strategies, or enhanced regional blood-brain barrier transport, might offer a promising approach to mitigate these impairments. Future research should focus on region-specific effects of insulin resistance and identify patient subgroups likely to benefit from insulin-based interventions. Integrating metabolic and neuropsychiatric perspectives may unlock new therapeutic avenues for AD.

*This work was supported by grants from NIH T32 (DK007260, to WC), the Steno North American Fellowship awarded by the Novo Nordisk Foundation (NNF23OC0087108, to WC), STI2030-Major Projects (2021ZD0202700, to HY), the National Natural Science Foundation of China (32241004, to HY), the Natural Science Foundation of Zhejiang Province of China (LR24C090001, to HY), Key R&D Program of Zhejiang Province (2024SSYS0017, to HY), CAMS Innovation Fund for Medical Sciences (2019-12M-5-057, to HY), Fundamental Research Funds for the Central Universities (226-2022-00193, to HY), the Non-profit Central Research Institute Fund of Chinese Academy of Medical Sciences (2023-PT310-01, to HY)*.
